# Lactate Metabolism and Signaling in Tuberculosis and Cancer: A Comparative Review

**DOI:** 10.3389/fcimb.2021.624607

**Published:** 2021-02-26

**Authors:** Dilara Kiran, Randall J. Basaraba

**Affiliations:** Metabolism of Infectious Diseases Laboratory, Mycobacteria Research Laboratories, Department of Microbiology, Immunology, and Pathology, College of Veterinary Medicine and Biomedical Sciences, Colorado State University, Fort Collins, CO, United States

**Keywords:** lactate, tuberculosis, immunometabolism, host-directed therapies, pathogenesis

## Abstract

Infection with *Mycobacterium tuberculosis* (Mtb) leading to tuberculosis (TB) disease continues to be a major global health challenge. Critical barriers, including but not limited to the development of multi-drug resistance, lack of diagnostic assays that detect patients with latent TB, an effective vaccine that prevents Mtb infection, and infectious and non-infectious comorbidities that complicate active TB, continue to hinder progress toward a TB cure. To complement the ongoing development of new antimicrobial drugs, investigators in the field are exploring the value of host-directed therapies (HDTs). This therapeutic strategy targets the host, rather than Mtb, and is intended to augment host responses to infection such that the host is better equipped to prevent or clear infection and resolve chronic inflammation. Metabolic pathways of immune cells have been identified as promising HDT targets as more metabolites and metabolic pathways have shown to play a role in TB pathogenesis and disease progression. Specifically, this review highlights the potential role of lactate as both an immunomodulatory metabolite and a potentially important signaling molecule during the host response to Mtb infection. While long thought to be an inert end product of primarily glucose metabolism, the cancer research field has discovered the importance of lactate in carcinogenesis and resistance to chemotherapeutic drug treatment. Herein, we discuss similarities between the TB granuloma and tumor microenvironments in the context of lactate metabolism and identify key metabolic and signaling pathways that have been shown to play a role in tumor progression but have yet to be explored within the context of TB. Ultimately, lactate metabolism and signaling could be viable HDT targets for TB; however, critical additional research is needed to better understand the role of lactate at the host-pathogen interface during Mtb infection before adopting this HDT strategy.

## Introduction

*Mycobacterium tuberculosis* (Mtb) is the leading cause of death by an infectious agent worldwide, with 10 million new cases and 1.2 million deaths due to tuberculosis (TB) disease in 2018 alone according to the most recent World Health Organization (WHO) Global Tuberculosis Report (WHO | Global tuberculosis report, 2019). While the death rate has steadily declined over the past twenty years, slowly approaching goals set forth by the WHO, the incidence of new cases has remained relatively constant. The current COVID-19 pandemic is also expected to have significant impacts on TB case survival rates across the globe. Current reports indicate that patients with TB have a higher likelihood of developing severe complications from SARS CoV-2 and have a higher risk of morbidity and mortality as a result of co-infection (Gao et al., 2020; Sy et al., 2020). Overlapping biological and social determinants of TB and SARS CoV-2 risk increases the risk of TB patients contracting the novel coronavirus (Udwadia et al., 2020). National lockdowns and the increased burden on healthcare systems as a result of global pandemic responses have hindered TB surveillance, treatment, and accessibility of TB patients to routine care and have the potential to further divert resources away from TB disease treatment and prevention efforts (Manyazewal et al., 2020; McQuaid et al., 2020; Udwadia et al., 2020). It has been estimated that the impacts of the pandemic could result in not only an increase in TB cases but also deaths, especially in areas of high TB burden, which could result in 20% more deaths compared to if there had been no pandemic (Udwadia et al., 2020; Hogan et al., 2020).

Aside from the global COVID-19 pandemic, there are many existing barriers that continue to contribute to high global TB incidence and which have slowed the progress toward a disease cure including the rise of multi-drug resistance (MDR), which further complicates antimicrobial treatment options. MDR strains of Mtb are resistant to the two first-line anti-TB drugs rifampicin and isoniazid and recent years have seen the development of extensively drug-resistant TB (XDR-TB) which is additionally refractory to fluoroquinolones and second-line injectable antimicrobial drugs (WHO | Global tuberculosis report, 2019). Current recommended treatment for drug sensitive Mtb involves 6 to 9 months of a multi-drug cocktail, which can cause severe side effects such as liver toxicity, peripheral neuropathies, and a variety of other complications (Tornheim and Dooley, 2019). Treatment duration is significantly extended with MDR or XDR cases, resulting in higher costs and patient compliance challenges. While direct observational therapy strategies and enhanced financial support can improve treatment compliance, the poor health care infrastructure and low socioeconomic status in countries with highest endemicity pose the greatest challenge (McLaren et al., 2016; Singh et al., 2019; Story et al., 2019). Bedaquiline and delamanid were recently approved to treat MDR and XDR TB cases; however, these were the first new TB drugs in over five decades and they still have issues with toxicity, absorption, distribution, metabolism, and excretion (Zumla et al., 2013; Hoagland et al., 2016; Evans and Mizrahi, 2018). Few viable alternatives exist within late-stage clinical development, and remaining gaps in knowledge of Mtb physiology and metabolism within the host hinders target identification (Zumla et al., 2013; Hoagland et al., 2016; Evans and Mizrahi, 2018). Current research investigating new treatment regimens or repurposing antimicrobials and other drugs also face challenges in translating findings from bench-top to bedside, with significant hurdles to bring a treatment from preclinical to clinical application (Dooley et al., 2019; Tornheim and Dooley, 2019).

An additional factor that complicates TB control is the co-occurrence of other infectious and non-infectious diseases. Diseases such as HIV/AIDS and type 2 diabetes mellitus (T2DM), which also have high incidence in endemic regions, augment individual susceptibility to Mtb infection and worsen clinical outcomes of those with TB (Marais et al., 2013; Bates et al., 2015). Specifically, T2DM accelerates the progression of TB disease, enhances the proinflammatory cytokine response, and results in more severe inflammation associated with Mtb infection (Podell et al., 2014). Additionally, it has recently been shown that individuals with vitamin A deficiency are at higher risk for developing TB even compared to HIV co-infection (Aibana et al., 2017). The interaction between multiple communicable and non-communicable diseases severely impacts already high-risk populations and further exacerbates an already complex chronic disease progression associated with active TB. The Bacillus Calmette-Guérin (BCG) vaccine, while widely used to lessen the severity of TB in children, does not prevent infection and has variable efficacy in adolescents and adults (Tang et al., 2016). Further, many individuals that are infected with Mtb may not show or have transient clinical signs, but can remain infected, which may reactivate decades later. It is still unclear what host or pathogen factors contribute to the development of latent TB and unfortunately, current diagnostic tests fail to accurately identify these at-risk patients (Alsdurf et al., 2016; Cohen et al., 2019). As a result, this review seeks to highlight the importance of alternative therapeutic strategies for combatting Mtb infection and specifically discusses targeting lactate metabolism and signaling as potential targets.

## Complexities of Granuloma Pathology

The nature of the TB granuloma microenvironment is of particular interest in the search for host-directed therapeutic targets. TB disease is characterized by granulomatous inflammation, which typically originates in the lungs as a consequence of aerosol exposure to Mtb, but which can disseminate and develop in other regions of the body (Turner et al., 2003; Orme, 2014; Orme and Basaraba, 2014). The early, classic TB granuloma is comprised of a central core of mostly macrophages that harbor intracellular bacilli but progresses to include a mixture of other inflammatory and immune cells including neutrophils and lymphocytes (Turner et al., 2003; Orme, 2014; Orme and Basaraba, 2014). Mtb is predominantly intracellular during early stages of infection (Hoff et al., 2011). As the disease progresses, lesions undergo varying levels of necrosis, fibrosis, and mineralization. In advanced lesions, Mtb is not only present intracellularly within macrophages, but is present extracellularly as a result of extensive necrosis of infected cells and tissue, which are particularly tolerant or resistant to antimicrobial drug treatment (Karakousis et al., 2004; Hartkoorn et al., 2007; Hoff et al., 2011; Ackart et al., 2014). Importantly, there is significant granuloma heterogeneity between and within individuals that is dependent upon both host and pathogen factors (Irwin et al., 2015; Lanoix et al., 2015; Lenaerts et al., 2015; Matty et al., 2015). Specifically, multiple, morphologically distinct granuloma types have been identified in mice, differing in their pathology, predominant immune cell type, bacterial burden, and impact on survival (Irwin et al., 2015). This inherent lesion complexity is difficult to model in animals given the species or strain differences in host response to experimental Mtb infection. For example, common laboratory mouse strains like C57BL/6 and BALB/c fail to develop hypoxic granulomas with central necrosis and mineralization following low-dose aerosol exposure, while these are common features in guinea pigs and non-human primates (Via et al., 2008). Other mouse strains, including the C3HeB/FeJ and the CBA/J, have gained popularity more recently given these models develop heterogeneous granulomas that include hypoxia, necrosis and occasionally mineralization (Driver et al., 2012; Harper et al., 2012; Major et al., 2013). In addition, novel strains of diversity outbred mice are increasingly being used to more closely mimic the genetic diversity of human hosts and thus responses to Mtb infection (Kurtz et al., 2020). Chronic TB disease is characterized by walling off of granulomas by fibrous connective tissue as a reflection of the ongoing attempts to heal damaged tissue (Gil et al., 2010; Orme, 2014; Orme and Basaraba, 2014). As a consequence, viable and non-viable bacilli become further isolated from immune surveillance and killing and persistent Mtb antigens continue to chronically stimulate the immune system. Based on this, there is an ongoing debate as to whether the granuloma is a protective structure, containing bacteria, thus limiting dissemination, or if the lesion is harmful, masking the bacteria from immune cell infiltrates, limiting blood supply and therefore effective drug delivery (Bold and Ernst, 2009; Cooper and Torrado, 2012; Orme et al., 2015). It is becoming increasingly clear that TB granulomas are dynamic structures, and that variability in granuloma architecture and composition dictates the microenvironment to which Mtb are exposed, which has significant implications for disease progression and therapeutic strategies.

## Rise of Host-Directed Therapeutic Strategies to Combat TB

To overcome the challenges to develop new, more effective antimicrobial drug treatments and to combat drug-resistant infections, host-directed therapeutic (HDT) strategies are being explored. HDT involves developing treatments or repurposing previously approved compounds to target the host rather than the pathogen. In this way, HDT can potentially modulate host responses to better combat Mtb infection when used alone or in combination with antimicrobial drugs. The applicability of HDT in the treatment of TB has been recently reviewed (Hawn et al., 2015; Kiran et al., 2015; Zumla et al., 2015; Wallis and Hafner, 2015; Wallis et al., 2016; Zumla et al., 2016). HDTs for TB have long included the use of corticosteroids for their anti-inflammatory properties, but more recently, targeting angiogenesis to improve vascular perfusion of TB lesions, and promoting tissue healing by modulating matrix metalloproteinase activity has been proposed (Kiran et al., 2015). Other drugs such as metformin, a biguanide that is used extensively in the treatment of type 2 diabetes mellitus (T2DM) have recently been explored as HDTs for TB. Metformin functions in part as an inhibitor of complex I of the electron transport chain and has been shown to restrict intracellular Mtb growth, induce mitochondrial reactive oxygen species production, reduce TB pathology, inflammatory cytokine expression, and decrease the risk of developing active TB in patients with T2DM (Lin et al., 2018; Lachmandas et al., 2019; Oglesby et al., 2019; Yew et al., 2019; Abinaya et al., 2020; Rodriguez-Carlos et al., 2020). Additionally, ours and other laboratories have recently demonstrated that metformin improves clinical disease outcomes in animal models chronically infected with Mtb. In addition to reducing the lung lesion and bacterial burden, metformin normalizes T cell metabolic homeostasis within guinea pigs chronically infected with Mtb (Frenkel et al., 2020).

One additional area of interest has been preserving immune cell metabolism under conditions of chronic antigenic stimulation as occurs in a wide variety of infectious diseases including TB (Moguche et al., 2017; McKinney and Smith, 2018). Cellular metabolism has been tightly linked to the development of depleted or exhausted immune cell phenotypes (Bengsch et al., 2016; McKinney and Smith, 2018). The field of immunometabolism is rapidly expanding as more and more metabolites and metabolic pathways are shown to regulate protective cellular immune responses in the pathogenesis and protection of both infectious and non-infectious diseases (Shi et al., 2016; Van den Bossche et al., 2017; Singer et al., 2018; Russell et al., 2019; Pålsson-McDermott and O’Neill, 2020).

## Immunometabolism as an HDT Target

A key finding at the interface of cell metabolism and disease progression was made by Otto Warburg in the 1950s (Warburg, 1956; Weinhouse et al., 1956; Koppenol et al., 2011). Under normoxic conditions, cells typically rely on oxidative phosphorylation and the mitochondrial electron transport chain to generate energy in the form of ATP (Warburg, 1956; Weinhouse et al., 1956; Koppenol et al., 2011). This oxygen dependent metabolic process can be compromised when oxygen tension drops. As a result, cells shift to rely on glycolytic metabolic pathways, which do not require oxygen, to generate ATP (Warburg, 1956; Weinhouse et al., 1956; Koppenol et al., 2011). While less efficient, glycolysis allows cells to survive and function under hypoxic microenvironments. This process generates lactate, which allows cells to regenerate NAD+ to maintain glycolytic flux (Hosios and Heiden, 2018; Walker and Tian, 2018). Warburg demonstrated that cancer cells are capable of a metabolic shift from oxidative phosphorylation to glycolysis, even in the presence of oxygen (Warburg, 1956; Weinhouse et al., 1956). This effect deemed “aerobic glycolysis” or “The Warburg Effect” has now been described in multiple disease processes and within activated immune cells (Potter et al., 2016; Schwartz L et al., 2017; Chen et al., 2018; Escoll and Buchrieser, 2018). Metabolic alterations that resemble The Warburg Effect have been described for infection with multiple intracellular bacterial pathogens, including *Brucella abortus*, *Legionella pneumophila*, and *Chlamydia trachomatis* (Ojcius et al., 1998; Czyż et al., 2017; Escoll et al., 2017). Contrary to Warburg’s original observations in cancer, it is now understood that mitochondrial metabolism does not shut down completely, but rather there is a relative increase in glycolytic metabolism (Palsson-McDermott and O’Neill, 2013; Lu et al., 2015; Danhier et al., 2017). However, metabolic reprogramming is considered a hallmark of cancer, and is a result of an interplay between adaptation to hypoxia, oncogene activation, loss of function of tumor suppressors, and altered signaling pathways (Hanahan and Weinberg, 2011; Vaupel et al., 2019). Within the TB field, there have been conflicting results about whether the Warburg Effect is induced or inhibited during Mtb infection, depending in part upon the disease model, Mtb strain, and methodology used during the studies (Shi et al., 2015; Gleeson et al., 2016; Lachmandas et al., 2016; Cumming et al., 2018; Shi et al., 2019; Cumming et al., 2020; Hackett et al., 2020; Phelan et al., 2020; Vrieling et al., 2020). The heterogeneity of granuloma lesions and presence of multiple granuloma types within and between individuals likely further complicates the metabolic microenvironment (Irwin et al., 2015). This has been the case in cancer, with recent evidence indicating that the tissue environment as well as genetic lesions dictate the metabolic profile of tumors, and that cancer metabolic reprogramming is not one-size-fits-all (Yuneva et al., 2012; Davidson et al., 2016).

To better understand this phenomenon, many have turned to study macrophages, where immune cell phenotype and function is related to metabolic phenotype. Classically activated M1 macrophages are characterized by a glycolytic metabolic phenotype and are functionally pro-inflammatory. They contribute to tissue damage and additional immune cell recruitment by the production of cytokines like IL-1β, IL-6, TNF-α, IFN-γ (Van den Bossche et al., 2017; Hobson-Gutierrez and Carmona-Fontaine, 2018; Wang et al., 2019). In contrast, M2 macrophages have an anti-inflammatory role, contributing to tissue remodeling and tissue repair by a metabolic phenotype that is more oxidative in nature (Van den Bossche et al., 2017; Hobson-Gutierrez and Carmona-Fontaine, 2018; Wang et al., 2019). There is significant metabolic heterogeneity within macrophages associated with TB granulomas, including activated, inflammatory macrophages with an M1 phenotype, lipid laden foamy macrophages with increased fatty acid metabolism, epithelioid macrophages, multinucleated giant cells, and macrophages that exhibit an M2 phenotype and play a role in granuloma architecture remodeling (Marakalala et al., 2018). The balance between M1 and M2 macrophage metabolic phenotypes can dictate chronic disease progression and maintenance of TB granulomas (Marino et al., 2015). However, recent work indicates that macrophage subsets may not be as stable or well defined as once described, with cells responding to a combination of stimuli and sometimes expressing M1 and M2 signatures simultaneously (Martinez and Gordon, 2014; Chávez-Galán et al., 2015). Relevant to TB, alveolar macrophages were found to have a hybrid phenotype, expressing both M1 and M2 surface markers in healthy individuals, and this flexibility was posited to be helpful in maintaining a balance between protective immunity and tolerance within alveoli (Mitsi et al., 2018).

## HIF-1α as a Transcriptional Regulator

In part, the regulation of this metabolic shift occurs at the transcriptional level by hypoxia-inducible factor 1 (HIF-1). HIF-1 is a transcription factor that globally regulates the cellular response to hypoxic stress (Wang and Semenza, 1995; Wang et al., 1995; Greijer et al., 2005). HIF-1 responsive genes are involved in angiogenesis, erythropoiesis, cell survival, and importantly, cellular metabolism (Balamurugan, 2016). Under normoxic conditions, the HIF-1α subunit is hydroxylated at proline residues by prolyl hydroxylases (PHDs) (Ivan et al., 2001; Jaakkola et al., 2001; Kaluz et al., 2006). This hydroxylation sequence tags HIF-1α for ubiquitination *via* the von Hippel-Lindau factor and subsequent proteasomal degradation (Ivan et al., 2001; Jaakkola et al., 2001; Kaluz et al., 2006). However, PHDs require oxygen as a cofactor for their activity. As a result, under conditions of hypoxia, HIF-1α is not hydroxylated and is not degraded (Ivan et al., 2001; Jaakkola et al., 2001; Kaluz et al., 2006). HIF-1α can thus accumulate in the cytoplasm and translocate to the nucleus where it dimerizes with the HIF-1β subunit (Jaakkola et al., 2001; Ivan et al., 2001; Kaluz et al., 2006). The active HIF-1 transcription factor then binds to hypoxia response elements in the genome to transcribe genes which regulate glycolysis, as well as the production and transport of lactate (Ivan et al., 2001; Jaakkola et al., 2001; Kaluz et al., 2006). The activation of HIF-1α has been shown to be a generalized response to infection (Werth et al., 2010).

Multiple studies have demonstrated that HIF-1α is expressed and active during Mtb infection (Shi et al., 2015; Braverman et al., 2016; Prosser et al., 2017; Baay-Guzman et al., 2018; Knight and Stanley, 2019; Resende et al., 2020). In zebrafish infection models using *Mycobacterium marinum*, HIF-1α stabilization enhances IL-1β expression, and helps clear infections *via* activating neutrophils (Elks et al., 2013; Ogryzko et al., 2019; Schild et al., 2020). Deletion of HIF-1α in myeloid cells accelerates granuloma necrosis and impairs host responses to *Mycobacterium avium* infection in mouse models (Cardoso et al., 2015). Interestingly, in mice deficient of HIF-1α only in the myeloid lineage of cells, deletion of HIF-1α in chronic infection resulted in more extensive inflammation in interstitial lung and caused earlier death, indicating HIF-1α coordinates myeloid cell responses to Mtb (Resende et al., 2020). Additionally, the metabolic shift to glycolysis has been observed in the context of *in vitro* macrophage Mtb infection models in the early stages of infection (Qualls and Murray, 2015; Shi et al., 2015; Gleeson et al., 2016; Shi et al., 2019).

**Figure 1:**
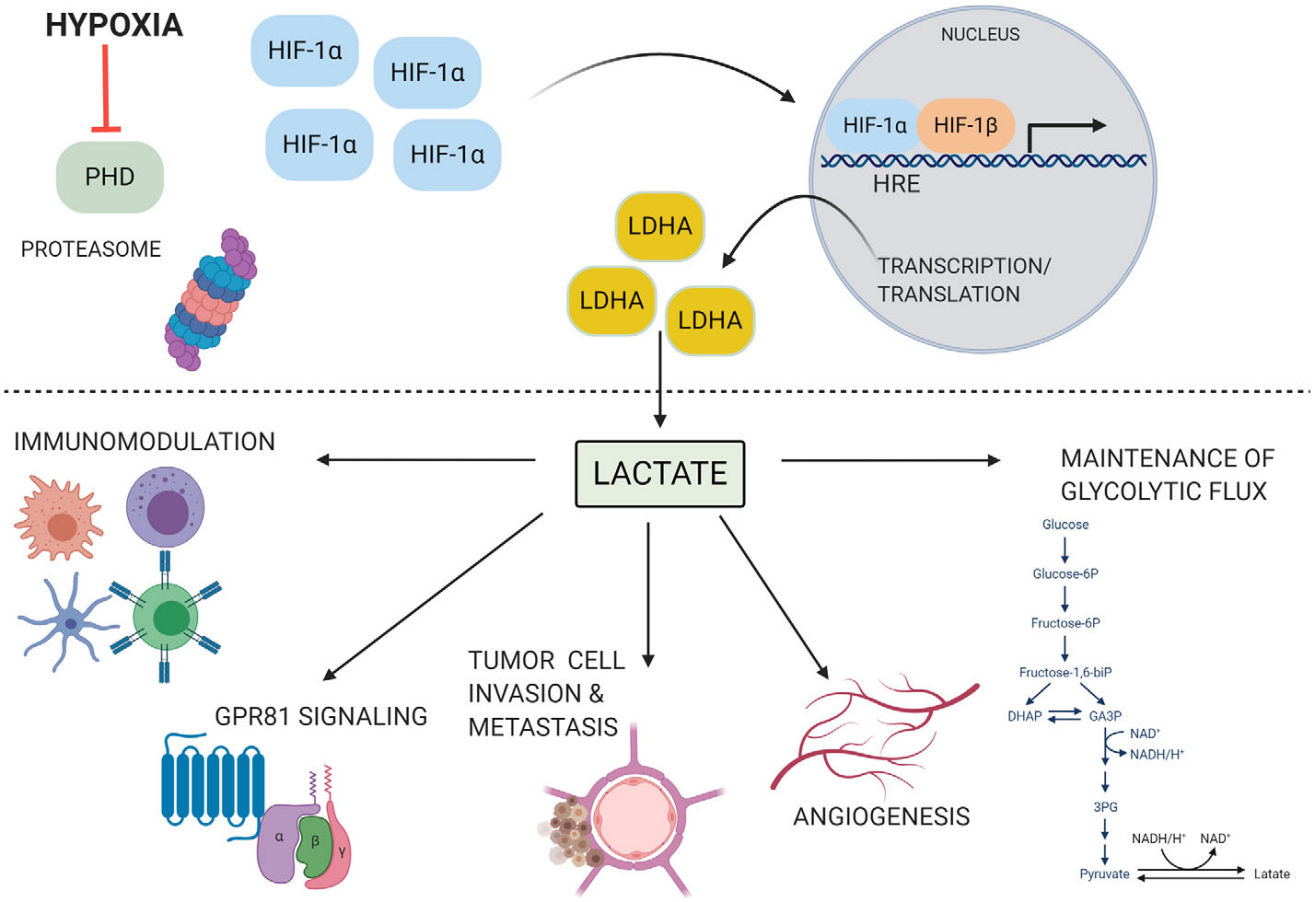
HIF-1α is stabilized and drives lactate production under conditions of hypoxia. Under conditions of hypoxia, low oxygen tension inhibits prolyl hydroxylase (PHD) activity. HIF-1α is not tagged for degradation and accumulates within the cytoplasm. It can then translocate to the nucleus, dimerize with the HIF-1β subunit, and bind to hypoxia responsive elements (HRE) on the genome. This initiates the transcription of a myriad of genes involved with the host adaptation to hypoxia, including lactate dehydrogenase A (LDHA). LDHA produces lactate, which can then go on to play a diverse role in many cellular processes, including angiogenesis, modulation of immune cell function, invasion and metastasis of tumor cells, signaling through G protein coupled receptor 81 (GPR81), and maintenance of glycolytic flux through regeneration of NAD+. These processes can contribute to disease pathogenesis in several contexts. Image produced using BioRender.com.

The downstream metabolic impact of HIF-1α expression is multifactorial. Pyruvate dehydrogenase kinase-1 is transcribed by HIF-1α, and prevents the conversion of pyruvate into acetyl-coA by phosphorylating the pyruvate dehydrogenase complex and increases extracellular lactate concentrations when active (Wigfield et al., 2008; Stacpoole, 2017). HIF-1α also controls expression of max interactor 1 and cytochrome c oxidase subunit 4, which further represses mitochondrial activity and decreases oxygen consumption during hypoxic conditions (Fukuda et al., 2007; Löfstedt et al., 2009). Not only does HIF-1α directly upregulate the transcription of enzymes involved in the glycolytic metabolic pathway, but it upregulates the production of lactate dehydrogenase A (LDHA) and monocarboxylate transporter 4 (MCT4) (Semenza et al., 1994; Firth et al., 1995; Semenza et al., 1996; Ullah et al., 2006; Rademakers et al., 2011). LDHA drives the production of lactate from pyruvate and MCT4 is responsible for lactate export from cells. Lactate itself, through its metabolism to pyruvate, can directly interfere with the proteasomal degradation of HIF-1α and carbohydrate response elements are present in several glycolytic genes (Walenta and Mueller-Klieser, 2004). Until recently, lactate was long believed to be a metabolic waste product. However, it has become increasingly clear that lactate serves a role in driving cellular metabolic changes that impact immune cell phenotypes, and that it can function as an extracellular signaling molecule to coordinate cellular responses to diverse microenvironments, as discussed below (**Figure 1**).

## The Importance of Lactate as a Metabolite

### Production and Accumulation of Lactate

The promoter for lactate dehydrogenase A (LDHA), which catalyzes the conversion of pyruvate into lactate, has a binding site for HIF-1α (Firth et al., 1995; Semenza et al., 1996; Cui et al., 2017). LDH isoforms are comprised of all A subunits (pyruvate → lactate), all B subunits (lactate → pyruvate), or a combination of both. While LDH is present in all tissues, the ratio of LDHA to LDHB subunits is tissue specific (Adeva et al., 2013). In some cancers, isoforms with only LDHA subunits have the highest efficiency at converting pyruvate to lactate and are correlated with increased HIF-1α and VEGF expression, increased tumor size, enhanced metastatic potential, and thus a poor prognosis (Koukourakis et al., 2003; Koukourakis et al., 2005; Kolev et al., 2008). However, some researchers do not believe the LDH enzyme distribution has an impact on the ability of cells to produce or use lactate, but rather affects the rate of the equilibrium of the reaction (Bak and Schousboe, 2017). LDH has a prognostic role in solid tumors, with high LDH associated with poor progression free survival and lower disease free survival (Petrelli et al., 2015). Additionally, serum LDH has been correlated with systemic inflammatory responses in patients with advanced pancreatic cancer (Yu et al., 2017). LDH has also been detected in elevated levels in bronchoalveolar lavage (BAL) fluid of active pulmonary TB patients, and BAL LDH correlates with increased serum LDH (Emad and Rezaian, 1999). Interestingly, sputum positive cases of TB show increased serum levels of LDH1, LDH2, and LDH3, which contain four, three, or two B subunits, respectively (Sharma et al., 2010). Increases in cerebrospinal fluid lactate have been shown to correlate to increases in severity of clinical stage for tuberculous meningitis (Siddiqi et al., 2018). This indicates that LDH levels and isoform specificity and lactate levels may also be diagnostic or prognostic indicators for pulmonary TB.

Much of what is known about the role of lactate comes from extensive reviews of the tumor microenvironment (San-Millán and Brooks, 2017; Pereira-Nunes et al., 2020). Lactate accumulates in the tumor microenvironment both in hypoxic solid tumors and during aerobic conditions due to the Warburg Effect, and malignant transformation is associated with increases in glycolytic flux (Walenta and Mueller-Klieser, 2004). Lactate enhances the release of macrophage pro-angiogenic factors, increases tumor metastatic activity, enhances collagen deposition, and worsens cancer prognosis (Crowther et al., 2001; Trabold et al., 2003). Lactate is also capable of diffusing from solid tumors and malignant cells to stimulate tumor associated fibroblasts, generating extracellular matrix rearrangement which allows for tumor cell migration and increases in tumor cell mobility (Walenta and Mueller-Klieser, 2004). Interesting, cancer cells that are more oxidative are sensitive to lactate signaling whereas more glycolytic cells are not and do not take up lactate and do not respond to exogenous lactate treatment (Pérez-Escuredo et al., 2015). While cancer research has led the effort in understanding the role of lactate in modulating carcinogenesis, much learned from this work can be applied in the realm of infectious disease. Early in Mtb infection, shifts to glycolytic metabolism are observed within macrophages leading to increased lactate production (Qualls and Murray, 2015; Shi et al., 2015; Gleeson et al., 2016; Osada-Oka et al., 2019; Shi et al., 2019) and high lactate concentrations are observable within granuloma lesions, reaching levels comparable to tumors (Somashekar et al., 2011; Somashekar et al., 2012).

### The Role of Lactate Shuttles

Lactate was largely considered to be a metabolic waste product of glycolysis until the 1980s, when the shuttling of lactate between cells and tissues was first described (Gladden, 2004; Brooks, 2009; Sun et al., 2017; Brooks, 2018; Ferguson et al., 2018; Brooks, 2020). One of the first described lactate shuttles was within muscle, with fast-twitch, glycolytic muscle fibers accumulating and releasing lactate and slow-twitch, oxidative muscle fibers importing and metabolizing lactate for energy (Brooks, 1985; Brooks, 1986; Brooks et al., 1999). Mitochondria have a role to play in shuttling lactate intracellularly, with LDH enzymes present in the mitochondrial intermembrane space and in the mitochondrial matrix, and monocarboxylate transporters (MCTs) present in the inner mitochondrial membrane to transfer lactate (Brooks et al., 1999; De Bari et al., 2004; Passarella et al., 2008). This is further evidenced by LDHB localizing to the mitochondria at greater densities than to other locations, and LDHA not localizing to the mitochondria (Chen et al., 2016). Additionally, mitochondrial LDH is highly expressed and more active in cancerous prostate cells than normal ones, contributing to the anaplerosis of TCA cycle intermediates to help fuel cancer progression (De Bari et al., 2010). However, some have demonstrated that lactate must be first converted to pyruvate before it can enter the mitochondrial matrix and have challenged the hypothesis that lactate can be directly oxidized by mitochondria (Jacobs et al., 2013; Passarella et al., 2014). Lactate shuttles have also been described between astrocytes and neurons, and mitochondrial LDH has been shown to play a role in allowing astrocytes to use lactate in the production of ATP *via* oxidative phosphorylation (Lemire et al., 2008; Magistretti and Allaman, 2018). Lactate has been demonstrated in multiple studies to be effectively metabolized by cancer cells and, in some instances, lactate is preferentially metabolized over other energy sources such as glucose (Kennedy et al., 2013; Faubert et al., 2017; Hui et al., 2017).

Monocarboxylate transporters (MCTs) are responsible for shutting lactate across membranes. Multiple MCTs exist, and cellular expression of MCTs depends on the physiological role of the cell (Halestrap, 2012; Felmlee et al., 2020). Highly glycolytic cells express MCT4, a low affinity transporter adapted to the export of lactate, while more oxidative cells predominantly express MCT1, which has a higher substrate affinity and is adapted for lactate import (Dimmer et al., 2000; Kirk et al., 2000). MCT1 has been demonstrated to be expressed in macrophages and increased upon LPS, TNF-α, or NO treatment (Hahn et al., 2000). The expression of MCT1 and MCT4 is associated with the expression of the chaperone CD147, which has transmembrane and cytoplasmic domains that interact with MCTs and help organize their distribution and localization on the plasma membrane (Kirk et al., 2000; Walters et al., 2013). CD147 has pro-tumor effects *via* its control of lactic acid transport, and knocking down CD147 reduces MCT1 and MCT4 expression and reduces the glycolytic rate by 50% (Le Floch et al., 2011). The invasive capability of human lung cancer cells was correlated with the expression of MCT1 and MCT4 and proliferation was reduced when these MCTs were inhibited (Izumi et al., 2011). Importantly, MCT4 is hypoxia-inducible, exhibiting 3-5 fold mRNA increases during hypoxia and showing significantly higher tissue expression in hypoxic areas as shown by co-localization with pimonidazole staining (Ullah et al., 2006; Le Floch et al., 2011; Rademakers et al., 2011). CD147 expression has also been demonstrated to be regulated by HIF-1α, promoting glycolysis and inhibiting tumor cell apoptosis (Ke et al., 2012; Ke et al., 2014). In some cancers, MCT1, MCT4, and CD147 have been identified as prognostic biomarkers which predicts poor patient survival (Latif et al., 2017; Luz MC de et al., 2017; Ruan et al., 2017). Within the context of Mtb infection, less is known about the presence and function of MCT1, MCT4, and CD147. CD147 has been demonstrated to be a marker of activated, highly suppressive regulatory T cells, and *in vitro* Mtb antigen stimulation expanded CD147 positive regulatory T cell subsets (Feruglio et al., 2015). Evaluation of mRNA from Mtb infected mouse lungs showed an increase in mRNA copy number of MCT4 over the course of infection, while MCT1 levels remained unchanged, indicating that high lactate efflux is an important host response to Mtb infection (Shi et al., 2015). Increased macrophage MCT4 was also confirmed recently *via* a transcriptional analysis of Mtb infected macrophages (Vrieling et al., 2020). MCT4 is also upregulated in macrophages upon stimulation of TLR2 and TLR4, through which Mtb antigens also stimulate macrophages responses, and knock-out of MCT4 can reduce macrophage proinflammatory cytokine production (Tan et al., 2015). MCT4 has also been shown to be present within the phagosomal membrane of *Mycobacterium bovis* BCG infected macrophages (Lee et al., 2010). Despite this, analysis of the dynamic role of MCTs in transporting lactate within the TB granuloma has not been described.

**Figure 2:**
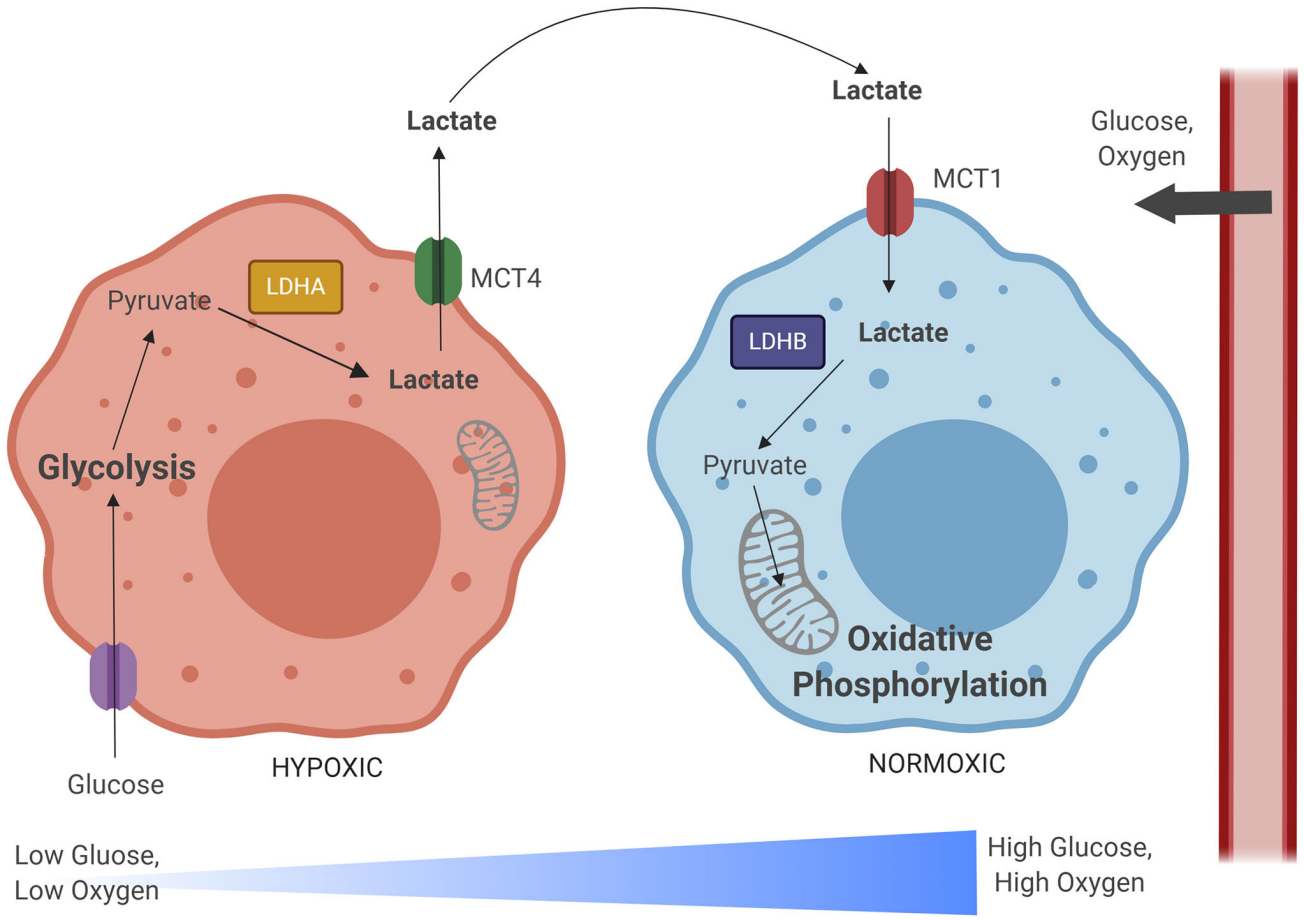
The lactate shuttle hypothesis illustrates a metabolic relationship between hypoxic and normoxic tumor cells. As glucose and oxygen diffuse out of tumor vascular supply, a gradient develops, with normoxic regions developing closer to the periphery and hypoxic regions developing within the lesion core. Hypoxic cells import glucose and rely on the glycolytic metabolic pathway to generate pyruvate. Pyruvate is converted to lactate by lactate dehydrogenase A (LDHA), and then exported in large quantities by monocarboxylate transporter 4 (MCT4). This lactate is imported by normoxic cells *via* monocarboxylate transporter 1 (MCT1). These normoxic cells preserve glucose that is needed for hypoxic cell metabolism and instead convert lactate back into pyruvate *via* lactate dehydrogenase B (LDHB). This lactate-derived pyruvate can then be used as fuel for the TCA cycle, oxidative phosphorylation, and mitochondrial respiration. Image produced using BioRender.com.

Combining knowledge about LDHs and MCTs, the lactate shuttle hypothesis posits that a metabolic symbiosis between hypoxic and oxidative tumor cells exists, wherein hypoxic, glycolytic cells produce lactate at high levels, and this lactate is transported to oxidative tumor cells, which convert lactate back into pyruvate to fuel their metabolism, and conserve glucose for hypoxic cells (Sonveaux et al., 2008) (**Figure 2**). Metabolically distinct cell populations should have unique signatures showing different expression levels of relevant metabolic enzymes and transporters, with glycolytic cells having increases in MCT4, LDHA, HIF-1α, while oxidative cells will have MCT1, LDHB, and will accumulate lactate (Nakajima and Van Houten, 2013). This hypothesis takes into account that solid tumors can be avascular, relying on diffusion and blood flow to obtain oxygen and glucose (Kim et al., 2011; Fadaka et al., 2017). Hypoxia, however, can initiate angiogenesis, creating a new capillary network to the tumor (Kim et al., 2011; Fadaka et al., 2017). Interestingly, models demonstrate that tissues have easier access to glucose within tumors with more chaotic microcirculation, and cells with high glycolytic capacity are more efficient at taking up limited glucose supplies, have higher lactate outputs, and maintain more phosphorylated glycolytic intermediates than cells with lower glycolytic capacity (Østergaard et al., 2013; van Beek, 2018). Glucose also appears to diffuse more readily in malignant tumors, such as adenocarcinoma and squamous cell carcinoma, than in non-malignant tissue (Zhao et al., 2010). This all supports the notion that peripheral cells in more oxidative environments preserve glucose for highly glycolytic, hypoxic cells. Metabolic modulations which characterize the TB granuloma are similar to those described within the tumor microenvironment. Therefore, these enzyme and transporter expression profiles, which occur in different regions of the tumor microenvironment, may directly apply to different regions of the granuloma microenvironment.

Indeed, a lactate shuttle has been proposed between astrocytes and microglia within the context of tuberculous meningitis (TBM), based on proton magnetic resonance based metabolomics of cerebrospinal fluid (CSF) samples of TBM-confirmed children (Mason et al., 2015). It is hypothesized that astrocytes respond to Mtb infected microglia by mobilizing glucose, increasing extracellular lactate, and increasing CSF lactate; this lactate is then used by microglia to fuel oxidative phosphorylation and reactive oxygen species production (Mason et al., 2015; Mason, 2017). In the presence of activating stimuli, such as LPS and IFNγ, microglia also convert glucose to lactate (Gimeno-Bayón et al., 2014). The role of lactate in the host response to TBM has been further highlighted, as high CSF lactate is a hallmark of TBM and all CSF lactate from TBM cases was found to be of the L-lactate form, indicating it was produced by the host rather than Mtb (Mason et al., 2016). Like macrophages present within pulmonary granulomas during TB disease, persistently activated microglia exhibit both neuroprotective and neurodestructive properties, contributing to nuances of host-pathogen dynamics in TBM (Hall et al., 1998; Giaume et al., 2007). Thus, further exploration of lactate shuttle dynamics and an evaluation of the expression of involved enzymes and transporters within the lung granuloma microenvironment is warranted.

### Lactate Facilitating Crosstalk Between Diverse Cell Types

Many cell types interact to shuttle and utilize lactate. Lactate modulates endothelial cells, and MCT1 driven lactate uptake in endothelial cells leads to significant increases in IL-8 production through the NFκβ-pathway (Végran et al., 2011). Additionally, lactate released from cancer cells *via* MCT4 stimulates IL-8 production, tumor angiogenesis, and tissue perfusion, supporting a model where tumor cell lactate can stimulate angiogenesis in endothelial cells (Végran et al., 2011). The impact of lactate on angiogenesis has been shown to be critical for the acceleration of healing in ischemic wounds, preventing muscle atrophy, and stimulating the production and release of angiogenic factors such as IL-8, bFGF, VEGF, and VEGFR (Xiong et al., 1998; Porporato et al., 2012). Crosstalk between cancer cells and endothelial cells *via* lactate release and MCT transport of lactate has also been demonstrated in glioma models (Miranda-Gonçalves et al., 2017).

In what has been deemed the “Reverse Warburg Effect,” cancer cells secrete hydrogen peroxide, which induces oxidative stress in cancer associated fibroblasts, causing them to undergo aerobic glycolysis in response, which produces large amounts of lactate that is then shuttled to tumor cells to fuel mitochondrial oxidative phosphorylation (Whitaker-Menezes et al., 2011; Fu et al., 2017; Koukourakis et al., 2017; Wilde et al., 2017). Further evidence of this is that MCT4, which exports lactate, is present in cancer associated fibroblasts and is up regulated by oxidative stress, and cancer cell MCT1 is up regulated when co-cultured with fibroblasts (Whitaker-Menezes et al., 2011; Fu et al., 2017; Koukourakis et al., 2017; Wilde et al., 2017). This metabolic synergy between cancer associated fibroblasts and cancer cells allows tumors that have been highly infiltrated by stromal cells to shift stromal metabolism to produce large quantities of lactate and export it *via* MCT4, and allow tumor cells to exploit the lactate produced, *via* MCT1 influx, and use it to fuel metabolism under glucose limited environments (Fiaschi et al., 2012; Curry et al., 2013). Lactate stimulates MCT1 expression in stromal cells, but reduces it in tumor cells, further suggesting that lactate is exported by tumor cells and taken up by stromal cells (Rattigan et al., 2012). Thus, the metabolic perturbations within the tumor microenvironment create spatial gradients based on differential sensitivity to lactate between cancer cells and stromal cells, such as tumor associated macrophages, and restricted perfusion contributes to these metabolic gradients, with cancer cells able to survive better in high lactate, hypoxic regions (Carmona-Fontaine et al., 2013). Spatial gradients also exist within the context of the TB granuloma, with different immune transcripts and proteomic profiles within necrotic, central regions as compared to the rim of the granuloma (Marakalala et al., 2016; Carow et al., 2019). Oxygen tension gradients and altered vasculature associated with granulomas also lead to restricted perfusion and central regions of hypoxia (Via et al., 2008; Datta et al., 2015; Datta et al., 2016). Based on these similarities between the granuloma and tumor microenvironments, it is likely that spatial differences in lactate metabolism and diverse crosstalk between heterogeneous cell types exists within the context of the TB granuloma. However, how differences in lactate metabolism and signaling manifest between different granuloma types, and their variable architecture and predominant immunologic cell type, is a remaining knowledge gap.

### Lactate Shuttling and Metabolism as a Therapeutic Target

Importantly, the lactate shuttle has been explored as a therapeutic target for diseases such as cancer, as lactate is a key regulator of carcinogenesis (Danhier et al., 2013; Doherty and Cleveland, 2013; Jones and Morris, 2016; Marchiq and Pouysségur, 2016; San-Millán and Brooks, 2017; Pivovarova and MacGregor, 2018). To exploit the need for glycolytic and oxidative tumor cells to shuttle lactate, researchers have explored MCT1 inhibition with α-CHC and have demonstrated that MCT1 inhibition can slow tumor progression and growth (Sonveaux et al., 2008). Lactate, released in the hypoxic tumor cell compartment, fuels the oxidative metabolism of cells that are in more vascularized, oxidized regions. This spares glucose for use in the hypoxic cells, which depend heavily on glycolysis. Inhibiting MCT1 thus induces a switch in oxidative cells from lactate fueled respiration to glycolysis; hypoxic cells die from glucose starvation, and cells that remain are more oxygenated and more susceptible to chemotherapies and radiation (Sonveaux et al., 2008; Mendoza-Juez et al., 2012). In malignant glioma, silencing MCTs decreases lactate efflux, and induces apoptosis and necrosis (Mathupala et al., 2004). Activated T cells rely on glycolysis for energy, produce increased amounts of lactate, and have higher MCT1 and MCT4 expression than resting cells; as a result, blocking lactate transport *via* MCT1 inhibits rapid phases of T cell division (Murray et al., 2005). Targeting MCT1 in endothelial cells can block the activation of HIF-1α by preventing lactate uptake and its conversion to pyruvate by LDHB, which subsequently competes with 2-oxoglutarate to inhibit PHDs needed to degrade HIF-1α (Sonveaux et al., 2012). This also blocks the downstream activation of angiogenesis. Inhibiting MCT1 can impair glycolysis and upregulate mitochondrial metabolism, improving the cellular bioenergetic state (Beloueche-Babari et al., 2017). Additionally, disrupting CD147, and therefore MCT1 and MCT4 expression, can sensitize lung cancer cells to biguanides like phenformin and metformin (Granja et al., 2014). Sensitization to phenformin could also be achieved by genetic knock out of MCT1 and CD147 (Marchiq et al., 2015). MCT1 knockdown is more effective under hypoxic conditions, resulting in larger decreases in lactate levels, cell biomass, cell invasion, and drastic reduction in breast cancer growth, and treatment with metformin could increase the response/efficacy of the MCT1 inhibition (Morais-Santos et al., 2015). The research combining MCT inhibition and biguanides is of interest for TB therapy, as metformin has been actively explored as a HDT (Lin et al., 2018; Lachmandas et al., 2019; Oglesby et al., 2019; Yew et al., 2019; Abinaya et al., 2020; Frenkel et al., 2020; Rodriguez-Carlos et al., 2020). However, inhibiting MCTs has yet to be explored in the context of Mtb infection. Based on the aforementioned accounts of increased MCT expression during Mtb infection, it is a viable area for future research.

Targeting LDHA has also been investigated as a way to increase the aerobic metabolism of pyruvate within the mitochondria, increasing reactive oxygen species production, oxidative damage, and cancer cell apoptosis (Doherty and Cleveland, 2013; Miao et al., 2013; Zhao et al., 2013; Gray et al., 2014). LDHA inhibition was found to increase apoptosis of A549 cancer cells (Xie et al., 2009). Inhibiting LDHA with compound FX11 decreased ATP levels, reduced mitochondrial membrane potential, increased oxidative stress linked to cell death, activated AMPK, and inhibited tumor xenograft progression in transformed human B cell lymphoma (Le et al., 2010). Simultaneously inhibiting LDHA *via* sodium oxamate and respiratory complex I *via* metformin depletes the cellular ATP pool, causing cancer cells to undergo metabolic catastrophe, leading to tumor cell growth arrest and cell death, leading to significant decreases in tumor size (Chaube et al., 2015). LDH inhibition *via* oxamate can also induce autophagy through inhibition of the AKT/mTOR pathway, reducing phosphorylation of Akt and downstream mediators mTOR and p70s6k (Zhao Z et al., 2015). Interestingly, if autophagy is inhibited, oxamate induces apoptosis and inhibits proliferation of cancer cells, indicating that the combination of oxamate with other inhibitors, such as those that prevent protective autophagy in cancers, are viable approaches (Zhao Z et al., 2015). Recent work demonstrates that inhibiting LDH *via* FX11 reduces the bacterial burden in Mtb infected mouse lungs, reduces the number of necrotic lesions, and potentiates isoniazid treatment (Krishnamoorthy et al., 2020). This may be due, in part, to the fact that Mtb is able to oxidize lactate *via* a quinone-dependent L-lactate dehydrogenase and, interestingly, lactate oxidation appears to be required for intracellular Mtb growth (Billig et al., 2017). Inhibiting macrophage production of lactate may therefore be a viable host-directed therapeutic strategy for combating Mtb infection. It has also been demonstrated that production of lactate through HIF-1α induced LDHA expression was critical for preventing intracellular pyruvate accumulation in Mtb infected macrophages (Osada-Oka et al., 2019). Interestingly, it was found that Mtb more efficiently used pyruvate over glucose to fuel its intracellular growth (Osada-Oka et al., 2019). LDHA may be required for the host to effectively restrict Mtb pyruvate use. Therefore, there may be dual roles for macrophage lactate production within the context of Mtb infection, as a result of a dynamic interaction between host and pathogen. This poses a unique challenge to the TB field that is not present in cancer. Additionally, the efficacy of these therapeutic strategies may be impacted by the type of granuloma, the stage of disease progression, and the host disease model. It is also important to consider the effects of modulating highly conserved metabolic pathways, such as lactate metabolism, on overall host health. For example, LDHA deficiencies have been shown to cause pustular psoriasis in some patients (Takeo et al., 2016). The relative impact of a therapeutic intervention on host cells must be accounted for when pursuing HDT for TB. Overall, a better understanding of metabolic fuel use between host and pathogen is critical for pursuing lactate shuttle inhibition as a therapeutic strategy for TB.

### The Immunomodulatory Effects of Lactate

Lactate also has distinct immunomodulatory effects (Morrot et al., 2018). Accumulated lactate in the tumor microenvironment, which can reach up to 40 mM concentrations, decelerates energy metabolism and induces suppressed phenotypes in infiltrating T cells by decreasing antigen-specific proliferation, and impairing T cell production of IL-1, IFN-γ, perforin and granzyme B (Fischer et al., 2007; Choi et al., 2013). Lactate inhibits CD4+ T cell chemotaxis, induces IL-17 production, and inhibits CD8+ T cell cytolytic activity (Haas et al., 2015). Macrophage LDHA is critical for macrophage dependent activation of antitumor CD8+ T cells and boosts IL-17 mediated immunity (Seth et al., 2017). IFN-γ producing CD4 T cells are predominant responders in anti-mycobacterial immunity; however, their role in TB pathogenesis is complex and these cells can stimulate neutrophil and macrophage mediated disruption of granuloma tissue architecture (Kumar, 2017). Cytolytic CD8 T cell function plays a critical role in Mtb infection, especially for long term control of infection (Lin and Flynn, 2015). Th-17 cells also play a diverse role in the pathogenesis of Mtb infection, particularly through their recruitment of neutrophils to the site of infection (Lyadova and Panteleev, 2015; Shen and Chen, 2018). Accounts of the relationship between T cells phenotype and lactate within TB are limited; however, the levels of lactate within the TB granuloma reach mM concentrations comparable to that of tumors, and LDHA expression colocalizes with CD3+ T cells in mouse lungs 30 days post infection (Somashekar et al., 2011; Shi et al., 2015). Based on this, it is likely that lactate may exert some of these described effects during Mtb infection. The level of lactate within the microenvironment may in part shape the type of granuloma lesion that develops, impacting therapeutic strategies. Specifically, the induction of IL-17 production and boost of IL-17 mediated immunity and simultaneous reduction in CD4+ and CD8+ T cell proliferation, infiltration, cytokine production, and effector activity *via* lactate may lead to described type II lesions, which are rapidly progressive and composed almost entirely of neutrophils (Irwin et al., 2015). Understanding how lactate concentrations vary within different TB granuloma types may shed insight into their immunologic phenotype and character.

Regulatory T cells (Tregs) are resistant to the suppressive effects of lactate on effector T cells, making these populations better able to survive in high lactate environments and assist in cancer immune evasion (Angelin et al., 2017). It has been shown that TB patients who have negative sputum smears post treatment have significantly lower levels of plasma IL-17, higher levels of plasma IL-10, and higher percentages of Tregs when compared to sputum positive individuals, potentially pointing to a role of Tregs in controlling TB progression (Xu et al., 2016). However, Tregs can antagonize the protective cellular immunity of Th1 and Th17 responses, which could result in loss of control of TB disease and the production of anti-inflammatory mediators by Tregs early in infection could enhance mycobacterial growth by suppressing the immune response (Brighenti and Ordway, 2017). In contrast, Tregs are also required to control inflammation, especially in prevention of autoimmune disorders. Interestingly, the regulation of the Th-17 versus Treg balance has been demonstrated to be a result of HIF-1α regulation, with active HIF-1α contributing to the differentiation of Th17 cells and a lack of HIF-1α enhancing Treg development (Dang et al., 2011; Shi et al., 2011). The timing of HIF-1α activation and its subsequent impact on increased lactate production may be key to determining whether an inflammatory or anti-inflammatory granuloma microenvironment forms, and impact the course of Mtb infection. A delicate balance of T cell regulation is key to the enhancement or resolution of TB pathology, and lactate accumulated within the granuloma may play a critical role in augmenting this balance.

NK cell production of granzyme B and perforin is also reduced in the presence of lactate, and NK cells treated with lactate express fewer activation receptors (Husain et al., 2013). Additionally, LDHA deficient tumors have significantly higher NK cell activity and fewer myeloid derived suppressor cells present (Husain et al., 2013). Tumor derived LDH can induce NK cell ligands on myeloid cells, which causes NK cells to down regulate associated receptors and impairs their anti-tumor activity (Crane et al., 2014). The high levels of lactic acid that inhibit CD8 T cell and NK cell activity is mainly through the inhibition of the transcription factor NFAT (Brand et al., 2016). NK cells are known to play a role in Mtb infection, *via* cytotoxic release of perforin and granzyme as well as through activation of macrophages and enhancing phagolysosomal fusion *via* IL-22 and IFN-γ production (Liu et al., 2017). Impaired NK cell activity *via* lactate-mediated mechanisms could reduce the phagolysosomal fusion capacity of macrophages, aiding the ability of Mtb to set up its intracellular residence in phagosomes. Decreased functionality of NK cells may prevent appropriate immune responses necessary to activate macrophages and induce mycobacterial killing, providing Mtb an additional advantage. Additional research is needed to explore the role of lactate in augmenting NK cell immunity within the context of TB.

Tumor derived lactate also significantly inhibits monocyte TNF-α secretion, impairs monocyte glycolytic flux, stimulates macrophage VEGF and TGFβ, up regulates monocyte IL-23 production, and inhibits the differentiation of monocytes to dendritic cells (Dietl et al., 2010; Goetze et al., 2011). Migration of monocytes is inhibited, while the migration of cancer cells is enhanced in lactate enriched environments, further contributing to tumor immune escape (Goetze et al., 2011). In the presence of lactate, GM-CSF activated macrophages are not able to produce pro-inflammatory cytokines and lactate facilitates tumor associated macrophages to undergo M2 polarization, even diverting differentiation of monocytes from dendritic cells toward M2 macrophages (Su et al., 2014; Zhao Y et al., 2015; Selleri et al., 2016; Ohashi et al., 2017). This would reduce antigen presentation and induce tissue remodeling. Similarly, Mtb is known to escape the immune system by reducing antigen presentation, and impairing dendritic cell maturation and migration, as well as by inhibiting phagolysosomal fusion and inhibiting apoptosis (Grace and Ernst, 2016; Ernst, 2018; Zhai et al., 2019). Lactic acid can also interfere with TLR signaling and delay the chemokine and cytokine response to LPS stimulated inflammation (Peter et al., 2015). Deleting LDHA in specific tumors reverses immunosuppression in the tumor microenvironment, likely through reduced PD-L1 and VEGF (Seth et al., 2017). The anti-inflammatory nature of lactate has also been demonstrated in models of intestinal inflammation, whereby treatment with lactate prevented TNBS induced colitis, reduced epithelial damage and edema, decreased the production of circulating IL-6, and prevented bacterial translocation to the liver (Iraporda et al., 2016). The levels of lactate within the TB granuloma have been shown to increase over the course of infection (Somashekar et al., 2011). An increase in M2 macrophage polarization, reduced antigen presentation, and impaired cytokine and chemokine responses as a result of high lactate concentrations may contribute to a shift in host responses during late stages of infection that promotes Mtb persistence and reduces pro-inflammatory macrophage responses. Lactate likely plays a critical role in regulating monocytes, macrophages, and dendritic cells critical for responses to Mtb infection and antigen presentation and, the amount of lactate present may influence the manifestation and progression of TB disease.

Ultimately, both the tumor microenvironment and the TB granuloma are formed and sustained by complex interactions between T cells, macrophages, and other immune cells. Both disease processes rely on immunosuppressive macrophage populations to prevent immune system elimination and appropriate infiltration of T cells and antigen presenting cells to elicit antigen-specific immune responses, as has been previously reviewed (Bickett and Karam, 2020). Thus, findings on the immunoregulatory role of lactate within the tumor microenvironment have merit to be translated to the context of TB disease. However, there are some differences between these disease processes that should be noted. First and foremost, TB disease is caused by an infectious agent, and an additional layer of complexity is added when the ways in which Mtb itself influences immune cell function are considered. Tumor cells are rapidly proliferating, whereas macrophages which harbor Mtb are not, and Mtb undergoes phases of slowed growth and persistence which contribute in part to latency of infection (Voskuil et al., 2003; Garhyan et al., 2015; Getahun et al., 2015; Hudock et al., 2017; Certo et al., 2020). Mechanistically speaking, lactate in the context of chronic inflammation has been shown to actively contribute to the retention of inflammatory T cells within tissues, and have different patterns of mitochondrial enzyme localization than in cancer (Pucino et al., 2019). Lactate can trigger intracellular signals that promote chronic inflammatory processes in inflammatory disease contexts, and has the potential to do this independently of HIF-1α (Lee et al., 2015; Pucino et al., 2019). CD4+ T cells in inflammatory environments that sense high lactate levels through a transporter known as SLC5A12 are pushed to differentiate into inflammatory subsets (Haas et al., 2015). Interestingly, there are some accounts which indicate that lactate results in diminished IFN-γ within the tumor microenvironment, but stimulates IFN-γ production in inflammatory environments (Brand et al., 2016; Peng et al., 2016; Certo et al., 2020). This would have significant implications for anti-Mtb immune responses. Variable granuloma types are also composed of different immunologic cell types at varied levels of activity, which impacts Mtb survival and severity of pathology (Irwin et al., 2015). The role of the immune system and goal of therapy is also different, in that for cancer, an abnormal population of human cells are being targeted, distinct from immune cells modulating the environment, whereas to combat Mtb, macrophages harboring Mtb themselves must be activated to enhance mycobacterial killing. Mode of cell death has an impact on Mtb disease, and thus selective killing of infected macrophages may not be a viable strategy, as is the case for selective targeting of tumor cells (O’Sullivan et al., 2007; Abebe et al., 2011; Lee et al., 2011; Srinivasan et al., 2014; Certo et al., 2020).

The differences and similarities between cancer and TB must be weighed accordingly when pursuing research questions regarding the role of lactate, and the potential for modulation of lactate metabolism as a therapeutic strategy, in either context (**Table 1**). However, given the limited number of mechanistic studies related to the role of lactate during Mtb infection and TB disease progression, the similarities pose interesting questions for further exploration. Overall, what is clear from the previous discussion is that lactate’s role in modulating immune cell bioenergetics and cellular phenotype points to more than just a role as an intermediary in metabolism, but points to critical roles in cell-cell signaling.

**Table 1:** A comparison of lactate metabolism and signaling in cancer and tuberculosis.

Lactate Metabolism & Signaling	Cancer	Tuberculosis	Outstanding Questions
Enhanced glycolysis, leading to increased lactate production and accumulation	• Hallmark of cancer (Hanahan and Weinberg, 2011; Vaupel et al., 2019) • Regulated by HIF-1α, which controls expression of LDHA (Ivan et al., 2001; Jaakkola et al., 2001; Kaluz et al., 2006) • LDHA is a poor prognostic indicator and correlated to increased tumor size (Koukourakis et al., 2003; Koukourakis et al., 2005; Kolev et al., 2008) • Lactate accumulates within the tumor microenvironment (Walenta and Mueller-Klieser, 2004)	• Glycolysis enhanced early during *in vitro* Mtb infection (Shi et al., 2015; Qualls and Murray, 2015; Gleeson et al., 2016; Shi et al., 2019) • HIF-1α expressed and active during Mtb infection (Shi et al., 2015; Braverman et al., 2016; Prosser et al., 2017; Baay-Guzman et al., 2018; Knight and Stanley, 2019; Resende et al., 2020) • Deletion of HIF-1α impacts granuloma necrosis, host responses, and chronic inflammation (Elks et al., 2013; Cardoso et al., 2015; Ogryzko et al., 2019; Schild et al., 2020) • LDHA increased in Mtb infection within macrophages and T cells (Shi et al., 2015) • Increased serum and BAL LDH within TB patients (Emad and Rezaian, 1999; Sharma et al., 2010) • Increased lactate within TB granulomas (Somashekar et al., 2011; Somashekar et al., 2012) • Increased CSF lactate in tuberculous meningitis correlates with disease severity (Siddiqi et al., 2018) • LDHA expression prevents pyruvate accumulation (Osada-Oka et al., 2019) • Mtb oxidation of lactate contributes to intracellular survival (Billig et al., 2017)	• Is HIF-1α a necessary host factor to constrain Mtb infection? Does it contribute to TB disease progression? • How does HIF-1α expression and activity vary across stages of Mtb infection, between granuloma types, and between disease models? • Does modulating host cell metabolism provide greater benefit to the host or Mtb, and how does the cell type targeted matter?
Lactate shuttles and cell-cell crosstalk	• Hypoxic, glycolytic cells produce lactate at high levels (via LDHA) and this lactate is exported to oxidative cells (via MCT4) to fuel their metabolism and conserve glucose (Sonveaux et al., 2008; Nakajima and Van Houten, 2013) • CD147 is a transmembrane chaperone for MCTs (Kirk et al., 2000; Walters et al., 2013) • Lactate stimulates endothelial cell production of IL-8 and enhances angiogenesis (Xiong et al., 1998; Végran et al., 2011; Porporato et al., 2012; Miranda-Gonçalves et al., 2017) • Reverse Warburg Effect between cancer associated fibroblasts and cancer cells creates spatial gradients based on sensitivity to lactate (Whitaker-Menezes et al., 2011; Fu et al., 2017; Koukourakis et al., 2017; Wilde et al., 2017)	• Astrocyte-microglia lactate shuttle proposed for tuberculous meningitis (Mason et al., 2015) • CD147+ T cell populations enhanced by Mtb antigen stimulation (Feruglio et al., 2015) • MCT4 expression increased during Mtb infection and enhanced by TLR2 and TLR4 signaling (Shi et al., 2015; Tan et al., 2015; Vrieling et al., 2020) • MCT4 expressed in the phagosome compartment of mycobacteria infected macrophages (Lee et al., 2010) • Angiogenesis implicated in increased Mtb dissemination (Polena et al., 2016)	• Do dynamic lactate shuttles exist within the TB granuloma microenvironment? • What cell types may be involved and do shuttling dynamics change between TB granuloma types, disease models, and during stages of Mtb infection? • How does lactate influence endothelial cells and fibroblasts present within the TB granuloma?
Lactate shuttle inhibition			• Is MCT inhibition, either alone or in combination with other therapies, beneficial in the case of TB? • Would LDH inhibition work better in combination with other inhibitors for Mtb infection? • LDHA inhibition limits the ability for Mtb to use host lactate, but can lead to pyruvate accumulation, which is efficiently metabolized by Mtb. Can these opposing dynamics be reconciled?
*MCT Inhibition*	• Slowed tumor progression and growth, blocked HIF-1α activation, and impaired glycolysis (Mathupala et al., 2004; Murray et al., 2005; Sonveaux et al., 2008; Mendoza-Juez et al., 2012; Sonveaux et al., 2012; Beloueche-Babari et al., 2017) • Decreased lactate efflux and induction of cell death (Mathupala et al., 2004) • Can sensitize cells to biguanides (Granja et al., 2014; Marchiq et al., 2015)	• No direct studies investigating MCT inhibition as a therapy for TB	
*LDHA Inhibition*	• Decreased ATP levels, reduced mitochondrial membrane potential, increased cell death, and inhibition of tumor progression (Xie et al., 2009; Le et al., 2010; Chaube et al., 2015) • Effects enhanced when combined with biguanides or autophagy inhibitors (Chaube et al., 2015; Zhao Z et al., 2015)	• Inhibition by FX11 reduces bacterial burden in Mtb infected mouse lungs, reduces necrotic lesions, and potentiates isoniazid treatment (Krishnamoorthy et al., 2020)	
Immunomodulation			• Do elevated lactate concentrations prevent infiltration and homing of T cells to the site of the TB granuloma? • Is lactate mediated enhancement of IL-17 immunity responsible for neutrophil predominant, rapidly progressive granuloma types? • Does lactate enhance Tregs and suppress immune cell function within the granuloma? Does this contribute to Mtb persistence? • Does the impact of lactate on immune cell function differ throughout the time course of TB and as lactate concentrations increase within the granuloma? • What is the role of increased lactate concentration on antigen presentation and impaired dendritic cell maturation and migration during Mtb infection? • Does the impact of lactate on immune cell types vary based on the immunopathologic phenotype of the TB granuloma?
*CD4+ T cells*	• Decelerated energy metabolism (Fischer et al., 2007; Choi et al., 2013) • Impaired IL-1 and IFN-γ production (Haas et al., 2015) • Inhibited chemotaxis (Haas et al., 2015) • Induction of IL-17 production (Haas et al., 2015)	• No direct studies investigating role of lactate on modulating CD4+ T cells in Mtb infection • IFN-γ producing T cells important for host response (Kumar, 2017) • Th-17 cells play role in neutrophil recruitment (Lyadova and Panteleev, 2015; Shen and Chen, 2018)	
*CD8+ T cells*	• Impaired perforin and granzyme B production (Fischer et al., 2007; Choi et al., 2013) • Inhibited cytotoxic activity (Haas et al., 2015)	• No direct studies investigating role of lactate on modulating CD8+ T cells in Mtb infection • CD8 T cells important for long term control of Mtb infection (Lin and Flynn, 2015)	
*Treg cells*	• Resistant to suppressive effects of lactate on effector T cells (Angelin et al., 2017)	• No direct studies investigating role of lactate on modulating Treg cells in Mtb infection • Higher IL-10 and higher T-reg percentages in sputum negative individuals post treatment (Xu et al., 2016)	
*NK cells*	• Impaired perforin and granzyme B production (Husain et al., 2013) • Expression of fewer activation receptors (Husain et al., 2013) • Inhibition of cytotoxic activity (Crane et al., 2014; Brand et al., 2016)	• No direct studies investigating role of lactate on modulating NK cells in Mtb infection • NK cell enhancement of phagolysosomal fusion *via* IL-22 and IFN-y and macrophage activation (Liu et al., 2017) • NK cell production of perforin and granzyme to control infection (Liu et al., 2017)	
*Monocytes/Macrophages*	• Inhibition of TNF-α secretion (Dietl et al., 2010; Goetze et al., 2011) • Impaired glycolytic flux (Dietl et al., 2010; Goetze et al., 2011) • Stimulation of VEGF and TGF-β (Dietl et al., 2010; Goetze et al., 2011) • Increased IL-23 production (Dietl et al., 2010; Goetze et al., 2011) • Inhibition of cell migration (Goetze et al., 2011) • Induction of M2 macrophage polarization (Su et al., 2014; Zhao Y et al., 2015; Selleri et al., 2016; Ohashi et al., 2017) • Interference with TLR signaling (Peter et al., 2015)	• No direct studies investigating role of lactate on modulating monocytes/macrophages in Mtb infection • Balance of M1 and M2 macrophages has an impact on granuloma progression and outcomes of disease (Marino et al., 2015)	
*Dendritic cells*	• Inhibition of monocyte differentiation into dendritic cells (Dietl et al., 2010; Goetze et al., 2011; Su et al., 2014; Zhao Y et al., 2015; Selleri et al., 2016; Ohashi et al., 2017)	• No direct studies investigating role of lactate on modulating dendritic cells in Mtb infection • Mtb reduces antigen presentation and impairs dendritic cell maturation and migration (Grace and Ernst, 2016; Ernst, 2018; Zhai et al., 2019)	
GPR81 signaling	• Higher GPR81 expression (Lee et al., 2016) • Enhanced tumor growth, metastasis, cell proliferation, and mitochondrial activity (Roland et al., 2014; Brown and Ganapathy, 2019) • Promotes malignant phenotype, cell migration, and angiogenesis (Lee et al., 2016) • Prevents antigen presentation (Brown et al., 2020) • Facilitates high rates of fatty acid synthesis (Stäubert et al., 2015) • Induces PD-L1 expression, leading to suppressed T cell function and immune evasion (Feng et al., 2017; Daneshmandi et al., 2019; Feichtinger and Lang, 2019) • Enhances chemoresistance (Wagner et al., 2017a; Wagner et al., 2017b)	• No direct studies investigating GPR81 expression or signaling during Mtb infection • Mtb relies on host fatty acids during infection (Singh et al., 2012; Lee et al., 2013) • PD-1 deficiency or blockade can increase TB susceptibility and severity (Lázár-Molnár et al., 2010; Tousif et al., 2011; Barber et al., 2019)	• Is GPR81 expressed at higher levels in TB granuloma tissue versus normal tissue? • Is GPR81 differentially expressed on different cell types within the TB granuloma? • How does GPR81 signaling augment Mtb infection and TB disease progression? • Would inhibition of GPR81 benefit or impair immune responses to Mtb?

## The Importance of Lactate as a Signaling Molecule

### Introduction to GPR81

Lactate is one of many metabolites which signals through G-protein coupled receptors (Offermanns, 2014; Husted et al., 2017; Offermanns, 2017; Ristic et al., 2017). Lactate was revealed to be an endogenous ligand for GPR81 through the discovery that GPR81 activation in adipose tissue suppressed lipolysis and mediated insulin-induced antilipolytic effects in an autocrine fashion (Lee et al., 2001; Cai et al., 2008; Liu et al., 2009; Ahmed et al., 2010). GPR81 is expressed in brown fat, white fat, kidney, liver, skeletal muscle, brain, and lung, among other tissues and expression profiles are similar across mouse and human tissue (Liu et al., 2009). Receptor structural residues are also conserved across species from humans to zebrafish (Kuei et al., 2011). GPR81 is coupled to a Gi subunit, which inhibits adenylyl cyclase, decreasing intracellular cAMP and causing protein kinase A (PKA) to be less active (Cai et al., 2008; Jeninga et al., 2009). Downstream signaling of GPR81 also activates the ERK1/2 pathway, PKC, PI3K, and Src kinases (Li et al., 2014). As previously mentioned, PKA has the ability to activate HIF-1 in a hypoxia-independent manner (Bullen et al., 2016; McNamee et al., 2016). Interestingly, since elevated cAMP and PKA signaling inhibits IFN-γ secretion by T cells during Mtb infection, activation of GPR81 may be a viable strategy to enhance Mtb directed immunity (Chung et al., 2008). Thiazolidinediones such as rosiglitazone can induce the expression of GPR81 through PPARγ, as a conserved PPARγ:retinoid X receptor-binding site is present in the promoter of the *Gpr81* gene (Jeninga et al., 2009). PPARγ has been demonstrated to have a role in Mtb infection, as knock-out of PPARγ in lung macrophages reduces Mtb growth and reduces granulomatous inflammation (Guirado et al., 2018). Mtb also limits host cell apoptosis through PPARγ induction (Arnett et al., 2018). Based on these observations, activation of PPARγ and subsequent induction of GPR81 expression may actually benefit Mtb by protecting its survival niche. Lactate signaling through GPR81, if present during Mtb infection, may have dual roles during infection, and future research to explore these hypotheses is warranted. Multiple synthetic agonists of GPR81 have been identified, some of which are commercially available, including 3-chloro-5-hydroxybenzoic acid and 3,5 dihydroxybenzoic acid (DHBA) (Dvorak et al., 2012; Liu et al., 2012; Sakurai et al., 2014; Geyer et al., 2015; Davidsson et al., 2020). These agonists have been investigated as alternative dyslipidemic agents to niacin, which signals through GPR109A and causes an undesired flushing reaction due to prostaglandin activation of Langerhans cells, but have yet to be actively explored as therapeutics for infectious disease applications (Dvorak et al., 2012; Liu et al., 2012; Sakurai et al., 2014).

### Diverse Roles of GPR81 Signaling

GPR81 plays a role in signal transduction across multiple body systems. Inflammation in adipose tissue initiated by LPS, zymosan, or turpentine stimuli resulted in the decreased expression of GPR81 (Feingold et al., 2011). GPR81 is also down regulated when adipocytes are exposed to macrophage derived inflammatory mediators *via* conditioned media (Rooney and Trayhurn, 2011). Further, lactate suppresses the induction of the NLRP3 inflammasome and IL1β *via* GPR81 activation, regulates TLR activation *via* interactions with arrestin B2 downstream of GPR81, and protects against inflammatory injury in pancreatitis and hepatitis models through GPR81 signaling (Hoque et al., 2014). Mtb infection has been demonstrated to inhibit NLRP3 inflammasome activation and IL-1β processing, and increased inflammasome activity has been associated with protection against active TB (Master et al., 2008; Briken et al., 2013; Souza de Lima et al., 2016). GPR81 has been demonstrated to be expressed by non-hematopoietic and immune cells such as macrophages and neutrophils, and these innate immune cells expressing GPR81 were critical in protecting mice from experimental colitis, and were involved in regulating the balance between IL-17 producing cells and Tregs (Ranganathan et al., 2018).

Within the CNS, GPR81 is concentrated along the plasma membranes of vascular endothelial cells, acting as a volume transmitter and serving as a feedback mechanism to counteract damage and save energy under hypoxic conditions (Bozzo et al., 2013; Lauritzen et al., 2014; Morland et al., 2015; Mosienko et al., 2015; Abrantes H de et al., 2019). GPR81 is highly enriched in leptomeningeal fibroblast like cells surrounding pial blood cells, and its activation stimulates cerebral VEGF and angiogenesis (Morland et al., 2017). However, in some conditions, such as ischemic brain injury, inhibiting lactate signaling through GPR81 can reduce ischemia induced apoptosis and provide neuroprotection, potentially indicating a dual role of lactate at low versus high concentrations (Shen et al., 2015). Importantly, within the context of the TB granuloma, vascularization and angiogenesis are important for enhancing oxygen delivery and drug delivery to central, hypoxic lesions. Angiogenesis has also been implicated in allowing for increased dissemination of Mtb, as inhibiting angiogenesis *via* VEGF inactivation abolishes mycobacterial spread (Polena et al., 2016). Thus, lactate’s role in endothelial cell signaling through GPR81 may augment TB pathogenesis.

GPR81 has been shown to play a critical role in cancer, where silencing GPR81 in tumor cells reduced tumor growth, metastasis, cell proliferation, and mitochondrial activity (Roland et al., 2014; Brown and Ganapathy, 2019). GPR81 expression is significantly higher in cancer tissue compared to adjacent noncancerous tissues and GPR81 promotes cancer aggressiveness, malignant phenotype, cell migration, and promotes amphiregulin transcription, contributing to angiogenesis (Lee et al., 2016). Tumor cell derived lactate can activate GPR81 on dendritic cells, and prevent the presentation of tumor specific antigens to other cells (Brown et al., 2020). Further, GPR81 signaling facilitates higher rates of fatty acid synthesis by inhibiting lipolysis, which allows for cancer cells to have increased viability and higher proliferation rates (Stäubert et al., 2015). Mtb is known to rely on fatty acids and augment host lipid metabolism during infection (Singh et al., 2012; Lee et al., 2013). Signaling through GPR81 has been shown to induce the expression of PD-L1, which can lead to suppressed T cell function and immune evasion in the tumor microenvironment, and blocking lactate production can improve PD-1 targeted immune checkpoint therapies (Feng et al., 2017; Daneshmandi et al., 2019; Feichtinger and Lang, 2019). PD-1 has been demonstrated to interfere with effector T cell function during Mtb infection, and blocking PD-1 can bolster IFN-y responses (Jurado et al., 2008). However, mice deficient in PD-1 have been shown to be very susceptible to Mtb infection (Lázár-Molnár et al., 2010; Tousif et al., 2011). Human clinical case reports also suggest that PD-1 blockade can increase the severity of TB (Barber et al., 2019). GPR81 activation stimulates the expression of ABC transporters which contribute to chemoresistance of cancer cells and GPR81 signaling enhances DNA repair mechanisms (Wagner et al., 2017a; Wagner et al., 2017b). These transporters may play a role in cellular resistance to antimicrobial therapies for Mtb. Additionally, GPR81 agonism can induce a vasoconstriction response, diverting blood flow away from the kidney and inducing hypertension across multiple species (Wallenius et al., 2017). Vasoconstrictive responses may impede blood flow to the TB granuloma, contributing to tissue hypoxia.

GPR81 plays a critical role in many processes that are important to the pathogenesis of TB. The anti-inflammatory responses stimulated by lactate activation of GPR81 may contribute to immune evasion by Mtb but may also be critical for controlling chronic inflammatory processes and limiting disease severity. The evaluation of the role of this receptor during Mtb infection has yet to be conducted, either *via* agonizing or antagonizing this receptor *in vitro* and *in vivo*, but GPR81 shows promise as a novel HDT target to modulate TB disease.

## Concluding Remarks and Perspectives

All and all, this body of literature paints a complex picture of the multitude of roles lactate plays as both a metabolite and signaling molecule during chronic, progressive disease. Importantly, it reveals a significant knowledge gap and lag within the field of Mtb research with respect to these mechanisms and pathways when compared to the field of cancer research. Mechanistic knowledge of the metabolic interactions occurring at the host-pathogen interface will aid in the development of host-directed therapeutic strategies that can augment cell metabolism, thereby shifting immune cell phenotypes to those that are better equipped to combat TB disease. In the context of Mtb, shifting macrophage metabolism and augmenting the granuloma microenvironment will better equip the host to resolve lesions and slow disease progression, potentially limiting the establishment of latent infections. Specifically, this review reveals promising new areas of investigation into the immunomodulatory roles of lactate within the granuloma microenvironment.

Increased glycolysis and lactate production by macrophages within the granuloma may serve to promote an Mtb survival niche, as lactate contributes to immune evasion and immunosuppression in the tumor microenvironment. The potential presence of active lactate shuttles between hypoxic cells and more normoxic cells within the granuloma microenvironment may serve as a target pathway for therapeutic intervention, as inhibiting this shuttle could limit the metabolic capacity of macrophages harboring Mtb, thus making the cellular environment less suitable for Mtb. In contrast, it is possible that the increased lactate production is a protective response which occurs by the host in the granuloma microenvironment, promoting M2 macrophage polarization, tissue remodeling and lesion repair. Additionally, anti-inflammatory signaling occurring *via* lactate through GPR81 may serve to reduce excessive inflammation and dampen disease progression. It is also important to note that host responses within tumor microenvironments and inflammatory environments, although similar in some respects, are not equivalent, and diversity in response exists between diseases of infectious and non-infectious inflammatory origin (Certo et al., 2020). This further exemplifies the need for the TB field to pioneer studies in this area.

**Figure 3:**
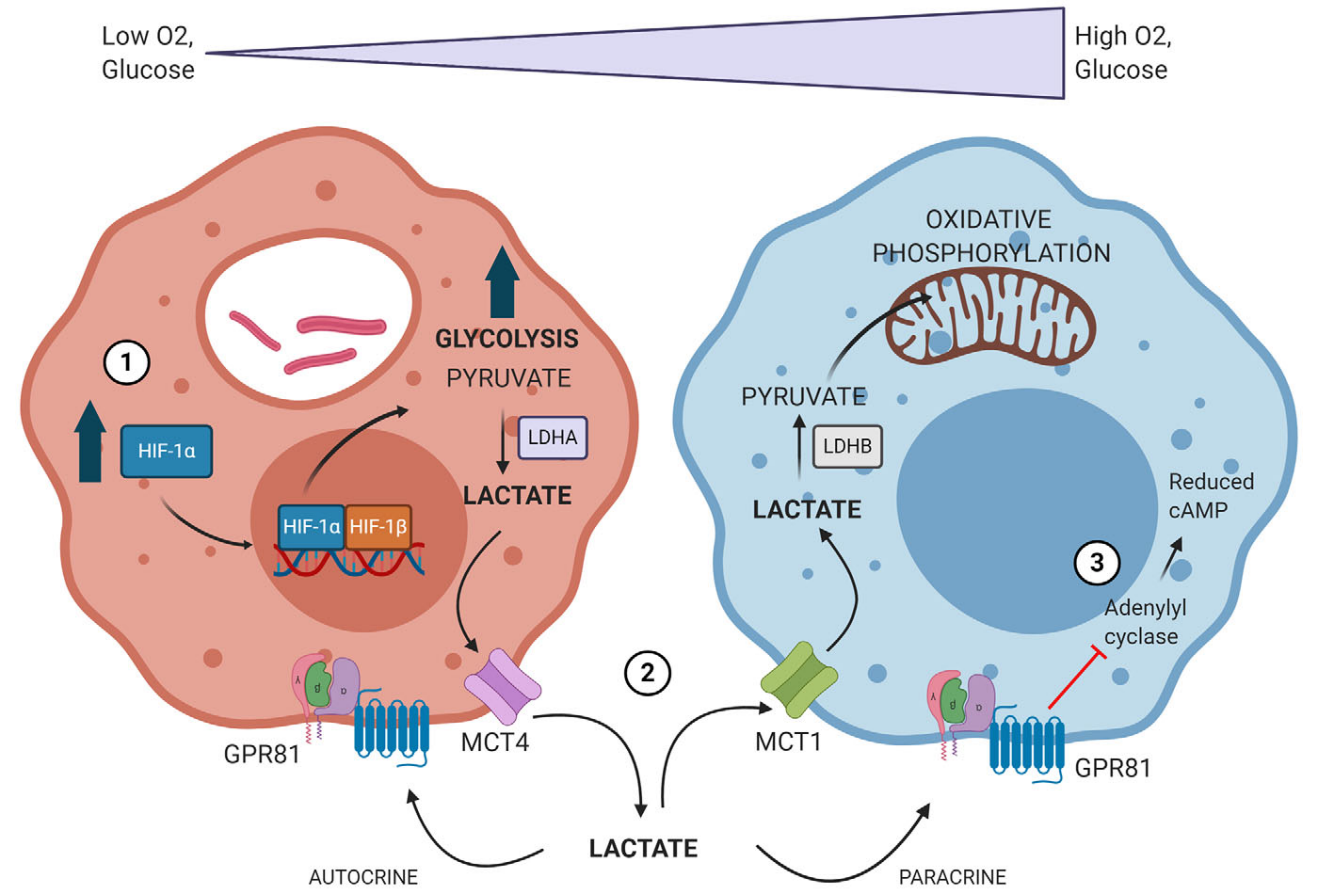
Proposed model of how Mtb infection drives changes in immune cell metabolism to establish its survival niche. 1) Mtb-infected macrophages accumulate stabilized HIF-1α, which subsequently translocates to the nucleus, initiating the transcription of genes related to glycolysis, lactate metabolism, and lactate transport. 2) The oxygen gradient present within TB granulomas yields hypoxic cells within the granuloma lesion core which rely heavily on glycolysis and produce large amounts of lactate. This lactate is produced *via* lactate dehydrogenase A (LDHA) and exported *via* MCT4. Lactate is then shuttled to more normoxic cells closer to the lesion periphery, where it is imported *via* MCT1 and converted back into pyruvate by lactate dehydrogenase B (LDHB). Lactate is then used preferentially over glucose as a source for pyruvate and can drive oxidative phosphorylation and mitochondrial respiration. 3) Lactate accumulating within the granuloma microenvironment can signal in both an autocrine and paracrine fashion through GPR81. GPR81 signaling inhibits adenylyl cyclase, reducing intracellular levels of cAMP. This initiates downstream signaling impacts that are anti-inflammatory and immunomodulatory. Image produced using BioRender.com.

Based on our knowledge and experience within the TB field, we have overarching hypotheses with respect to the role lactate plays in Mtb infection. Stabilization of HIF-1α during Mtb infection leads to a shift toward macrophage glycolytic metabolism, which is followed by production of large quantities of lactate. This lactate can then be shuttled to more oxidative regions of the granuloma microenvironment to preserve glucose for hypoxic cells. Lactate within the granuloma microenvironment can then serve as both an autocrine and paracrine anti-inflammatory, immunomodulatory signal *via* GPR81, augmenting the immune response to Mtb infection (**Figure 3**). However, it is important to note that due to the relatively little exploration into this field as of yet, we still do not know if 1) promoting or inhibiting lactate metabolism and signaling will be more beneficial, 2) if the consequences of intervening in these pathways remain the same throughout disease progression time course, or 3) if augmenting these pathways could be beneficial prior to disease onset. Ultimately, investigating the role of lactate as both a metabolite and signaling molecule during Mtb infection will lend critical insight into disease pathogenesis and host-pathogen interactions within the granuloma microenvironment.

## Author Contributions

DK and RB conceptualized this work. DK wrote the primary manuscript and RB provided edits. DK and RB both approved the final submitted version. All authors contributed to the article and approved the submitted version.

## Funding

This work was supported by funding from the National Institutes of Health, grant numbers 1F30OD024647, 1U19AI111224-01, 1R21AI107254, and 1R01AI106733. Funding was also provided by the Catalyst for Innovative Partnerships funding for ‘The Center for Metabolism of Infectious Diseases Accelerator’ by the Office of the Vice President for Research, Colorado State University.

## Conflict of Interest

The authors declare that the research was conducted in the absence of any commercial or financial relationships that could be construed as a potential conflict of interest.
